# Janus kinase inhibitor treatment for inflammatory diseases: excess or no excess risk of venous thromboembolism?

**DOI:** 10.1016/j.rpth.2024.102667

**Published:** 2024-12-31

**Authors:** Yachar Dawudi, Samuel Benarroch, Hélène Helfer, David M. Smadja, Isabelle Mahé

**Affiliations:** 1 Internal Medicine Department, Hôpital Louis-Mourier, Assistance Publique - Hôpitaux de Paris, Colombes, France; 2 Hematology Department, European Georges Pompidou Hospital, Assistance Publique - Hôpitaux de Paris, Paris, France; 3 Université Paris Cité, Paris, France; 4 INSERM Cardiovascular Research Center, Team « Endotheliopathy and Hemostasis Disorders », Paris, France; 5 Investigation Network On Venous Thrombo-Embolism (INNOVTE) - French Clinical Research Infrastructure Network, Saint-Etienne, France

**Keywords:** antirheumatic agents, autoimmune diseases, Janus kinase inhibitors, rheumatic diseases, venous thromboembolism, venous thrombosis

## Abstract

Janus kinase inhibitors (JAKis) have revolutionized the treatment landscape for various inflammatory and autoimmune diseases since their introduction in 2012. The expanded indications of JAKis have raised concerns about the associated risk of thrombosis, venous thromboembolic events (VTEs), and arterial thrombosis. This literature review examines studies reporting the risk of VTEs associated with JAKis in patients with inflammatory diseases. Phase I to III trials showed no increased risk of VTEs. However, these studies were not designed to detect adverse events such as VTEs. The pharmacovigilance data indicated that the frequency of VTE reports was higher than that of other adverse events. An increased risk of VTEs was also observed in the ORAL Surveillance study, a randomized, noninferiority, postmarketing phase IV safety study comparing tofacitinib with anti-tumor necrosis factor in patients with rheumatoid arthritis. However, limitations have to be acknowledged: pharmacovigilance data are declarative and subject to bias, VTE was a secondary outcome in the ORAL study, with noncomparable VTE risk factors between groups and increased thrombosis risks only at high doses of tofacitinib. Nevertheless, these data have led regulatory organizations such as the Food and Drug Administration and the European Medicines Agency to issue precautionary measures regarding the use of JAKis in inflammatory diseases. Most well-conducted real-life studies are in rheumatoid arthritis and do not confirm an excess of VTE risk associated with JAKis. Considering those conflicting results and limitations, future research should focus on specific indications and patient profiles, taking into account the complex interaction between drug treatment and underlying disease activity, to be able to draw definite conclusion about the VTE risk associated with JAKis.

## Essentials

• Janus kinase inhibitors (JAKis) are widely used to treat inflammatory diseases.
• JAKis associated risk of venous thromboembolic events is unclear.
• This review examines the data reporting on the venous thromboembolic risk on JAKis.
• Available data are conflicting and inconclusive.
• There is a need for targeted studies including disease activity, patient profile, JAKi type.

## Graphical abstract

**Figure:**
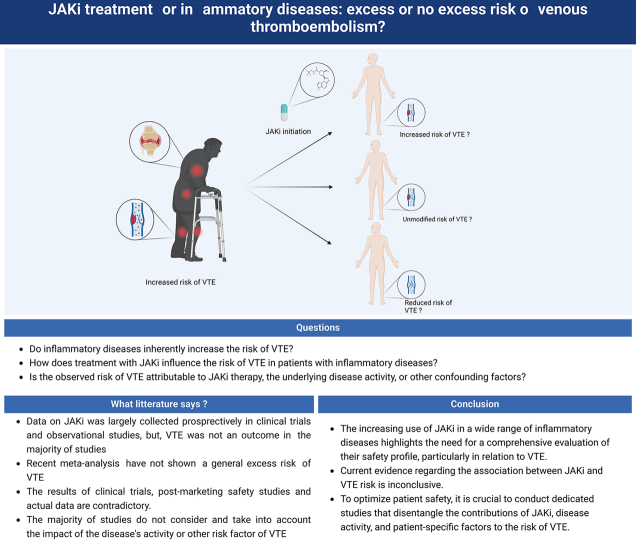

## 1 Introduction

Janus kinase inhibitors (JAKis) represent a novel class of orally administered molecules. Initially used in hematology to treat myeloproliferative disorders, they were introduced in 2012 as a second-line treatment for rheumatoid arthritis (RA). Since then, their range of indications in inflammatory diseases has significantly expanded. Today, they are prescribed in patients with RA, psoriasis with skin or rheumatic involvement, ankylosing spondylitis, inflammatory bowel diseases such as ulcerative colitis or Crohn’s disease, and atopic dermatitis (AD) [1]. To date, 5 JAKis are prescribed for inflammatory diseases management: baricitinib, abrocitinib, filgotinib, upadacitinib, and tofacitinib. The pathophysiological data are conflicting. JAKis target the JAnus tyrosine Kinase (JAK) and signal transducer and activators of transcription (STAT) signaling pathway, which is involved in signal transduction and transcription activation. This pathway is crucial for various cytokines and growth factors, playing a key role in hematopoiesis, inflammation, and immune functions. In view of the inhibition of the signaling pathway, side effects have emerged, such as herpes zoster infection, cytopenias, or even cancers in comparison with anti-tumor necrosis factor (anti-TNF) in inflammatory diseases, and more recently acne, whatever the active disease, including AD [[2], [3], [4]].

Moreover, concerns have emerged regarding the risk of thrombotic complications associated with JAKis. From a pathophysiological standpoint, inhibiting the JAK-STAT pathway appears to reduce endothelial prothrombotic activity [5]. However, in RA, JAKis also appear to increase cytokine production associated with immunothrombosis and delay clot lysis in active RA [6]. Therefore, the inhibition of the JAK-STAT pathway may paradoxically increase the risk of immunothrombosis and prothrombotic complications in conditions such as active RA. While some studies suggest a potential association between the use of JAKis and the occurrence of venous thromboembolic events (VTEs), other studies, including several meta-analyses, challenge this hypothesis. Despite these conflicting findings, warnings and precautionary recommendations for the use of JAKis in inflammatory diseases have been issued, impacting clinical practice and eligibility to treatment, and discontinuation in case of VTE [7,8], especially concerning the administration of high doses in patients considered to be at higher risk such as men over the age of 65 [9,10]. However, the literature on the JAKi-related VTE risk remains controversial. The aim of our literature review is to examine studies reporting VTE in patients treated with JAKis in the context of inflammatory diseases. This narrative review is based on a PubMed search, focusing on studies relevant to the thromboembolic risks associated with JAKis.

## 2 Venous Thromboembolic Risk Varies According to the Initial Pathology

Inflammatory diseases are known to be independent risk factors for VTE, although the associated increased risk varies according to the considered pathology. For example, patients with inflammatory bowel disease have an increased risk of deep vein thrombosis (DVT) or pulmonary embolism (PE) that is 3 to 4 times greater than in the general population [11,12]. This increased risk is all the more marked during the active phases of the disease, with hazard ratios (HRs) of 8.4 in the relapse phase compared with 2.1 in remission [11]. Other risk factors for VTE also appear to considerably further increase this risk [11,12]. Although the increased risk of VTE in RA is less pronounced than in inflammatory bowel disease, it is still 1.3 to 2 times higher than in the general population [13,14]. This risk is also associated with disease activity, with a higher HR during the first year following diagnosis compared with follow-up, when the disease is more controlled (HR at 1.6 vs 1.3 respectively) [13]. For AD, traditionally less associated with a risk of VTE, recent studies have demonstrated a moderate excess risk of VTE, about 1.1 to 1.3 times that of the general population [15,16]. However, the risk of VTE is less in AD than in other inflammatory diseases, with no apparent correlation with disease activity [17]. However, certain associated risk factors, such as age, appear to further increase this risk [16]. In conclusion, inflammatory diseases confer an increased risk of VTE. Reducing disease activity appears to mitigate this risk, while inadequate control tends to exacerbate it. Consequently, the interpretation of the role of JAKi treatment initiation in the occurrence of VTE in the context of uncontrolled disease is complex. There is a risk of overlooking the treatment’s role itself or misinterpreting a direct association between the treatment and the increased VTE risk. Given the varying inflammatory profiles of the diseases for which JAKi is indicated and the diverse patient profiles, it is crucial to consider the data separately based on the specific disease rather than adopting a global approach, due to these confounding factors.

## 3 Phase I, II, and, III Studies

The published literature on JAKis is rich and diverse, including multiple types of studies. The first clinical studies published on this subject were phase I studies, followed by phases II and III, totaling 62 studies (Supplementary Table S1). The characteristics of these studies, classified by indication, as well as the number of VTEs reported, are presented in Table 1. The majority of the studied molecules were tofacitinib at 31% (19/62), followed by upadacitinib at 24% (15/62), baricitinib at 16% (10/62), and filgotinib at 11% (7/62). All of these studies had a double-blind design and a control group. A large number of clinicobiological data were prospectively collected. None of these studies reported an increased risk of VTE in the JAKi groups compared with the control group. Eighty percent of the studies reported no cases of VTE in the JAKi group, while 20% mentioned 1 or 2 VTEs during the study. However, these studies were not primarily designed to assess treatment-related adverse events, and VTE was only systematically investigated in 29% of the studies; consequently, cases of VTE could have been missed. In addition, the limited number of patients (median 128 in the JAKi treatment groups and 111 in the control groups) and the relatively short follow-up period (median 12 weeks) did not allow rare and/or long-term adverse events to be identified. It should also be noted that the VTEs reported in these studies occurred during relapses of the disease. The different selective inhibition across JAKis leads to have a specific attention to delayed time to the occurrence of VTE after JAKi initiation [18]. The delay between the JAKi initiation and the occurrence of VTE was not specified. With the emergence of the various JAKis on the market and the extension of indications, numerous meta-analysis [3,[19], [20], [21], [22], [23], [24], [25], [26], [27], [28], [29]] have been published, including 4 published between January 2023 and January 2024 based on phase I to III studies [3,21,25,30]. Despite the large number of studies and patients, these recent meta-analyses, based on clinical studies, have not shown a general excess risk of VTE for all JAKis, regardless of the dosage used or the pathology for which JAKi is indicated. However, for the reasons mentioned above, it is difficult to conclude that there is or not an increased risk of VTE associated with JAKis on the basis of these studies alone. Furthermore, although randomization considerably reduces confounding bias, the strict inclusion and exclusion criteria, in particular the exclusion of patients with some VTE risk factors such as personal history of thrombosis (Supplementary Table S1), may limit the extrapolation of results to the population as a whole. This strict selection of patients could mask complications that only occur in particular types of patients.

**Table 1:** Summary of phase I to III studies of JAKis in inflammatory diseases.

Characteristic	Ankylosing spondylitis^a^ (*n* = 4)	Atopic dermatitis^a^ (*n* = 14)	Inflammatory bowel disease^a^ (*n* = 8)	Plaque psoriasis^a^ (*n* = 7)	Psoriatic arthritis^a^ (*n* = 6)	Rheumatoid arthritis^a^ (*n* = 20)	Systemic lupus erythematosus^a^ (*n* = 3)	Overall^a^ (*N* = 62)
Study design:								
*Phase I RCT*	0	0	0	0	0	0	1/3	1/62
*Phase II RCT*	2/4	3/14	4/8	5/7	2/6	3/20	2/3	21/62
*Phase III RCT*	2/4	11/14	4/8	2/7	4/6	17/20	0	40/62
Duration (wk)	13	16	8	12	14	18	12	12
Sample JAKi group	76	137	69	88	119	191	20	128
Sample control group	76	120	71	88	118	160	10	111
VTE researched specifically	2/4	2/14	3/8	5/7	1/6	4/20	1/3	18/62
JAKi arm VTE events	1/4	2/14	3/8	0/7	2/6	4/20	1/3	13/62
Control arm VTE events	0	1/14	0/8	0/7	2/6	5/20	0	8/62
Studies showing increased risk of VTE	0	0	0	0	0	0	0	0

JAKi, Janus kinase inhibitor; RCT, randomized controlled trial; VTE, venous thromboembolic event.

a *n*/*N* for categorical variables; median for continuous variables.

### 3.1 Pharmacovigilance studies

Pharmacovigilance, by recording adverse events under real-life conditions, allows for the detection of issues that may have been missed in phase I, II, and III clinical trials. Since the introduction of JAKis for inflammatory diseases in 2012, cases of VTE have been reported in pharmacovigilance databases. This has led to pharmacovigilance studies conducted in the United States using the Food and Drug Administration (FDA) Adverse Event Reporting System [31,32] and at an international level through the World Health Organization pharmacovigilance system [33]. These 2 studies report an increased risk of VTE associated with the use of JAKis compared with other adverse events. This assessment is based on the reporting odds ratio (ROR) and information components (ICs), 2 measures used in pharmacovigilance. The ROR compares the frequency of a specific adverse event, like VTE, for a particular drug relative to other reported events, where an ROR greater than 1 suggests a higher-than-expected reporting rate for that event. The IC, a Bayesian statistical measure, indicates the strength of association between a drug and an adverse event, with a positive IC suggesting that the event is reported more frequently than expected for the drug in question. In both cases, VTE reports for JAKis were significantly higher, pointing to a potential VTE risk [31,32]. VTEs included PE, DVT of the lower limbs, or more atypical thromboses such as portal thrombosis. Reported VTEs appeared to occur more frequently at the time of drug introduction, mainly in older men. The signal remains unchanged according to the JAKi studied, prescribed in a context of inflammatory disease (not specified) [32]. The number of VTEs reported by the FDA in patients treated with JAKis increased sharply between 2012 and 2021, and during this period, the number of FDA approvals for JAKis also increased, particularly during the treatment of inflammatory diseases (not specified) (Figure). This increase in JAKi indications may contribute to explain the increase in VTE reported. Nevertheless, the proportion of VTEs in relation to other reported adverse events became increasingly substantial, rising from 0.08% in 2012 to 30% in 2021 [32]. In addition, other signals suggesting a possible increase in cardiovascular risk and the development of cancers were also identified [33,34]. However, pharmacovigilance data are based on event reporting, which can be significantly biased. Factors such as awareness of the drug or potential adverse reactions can influence reports, complicating data interpretation. While pharmacovigilance is essential, it is challenging to draw definitive conclusions from its findings alone. Therefore, it is crucial to consider this information alongside clinical studies and real-world evidence.

**Figure:**
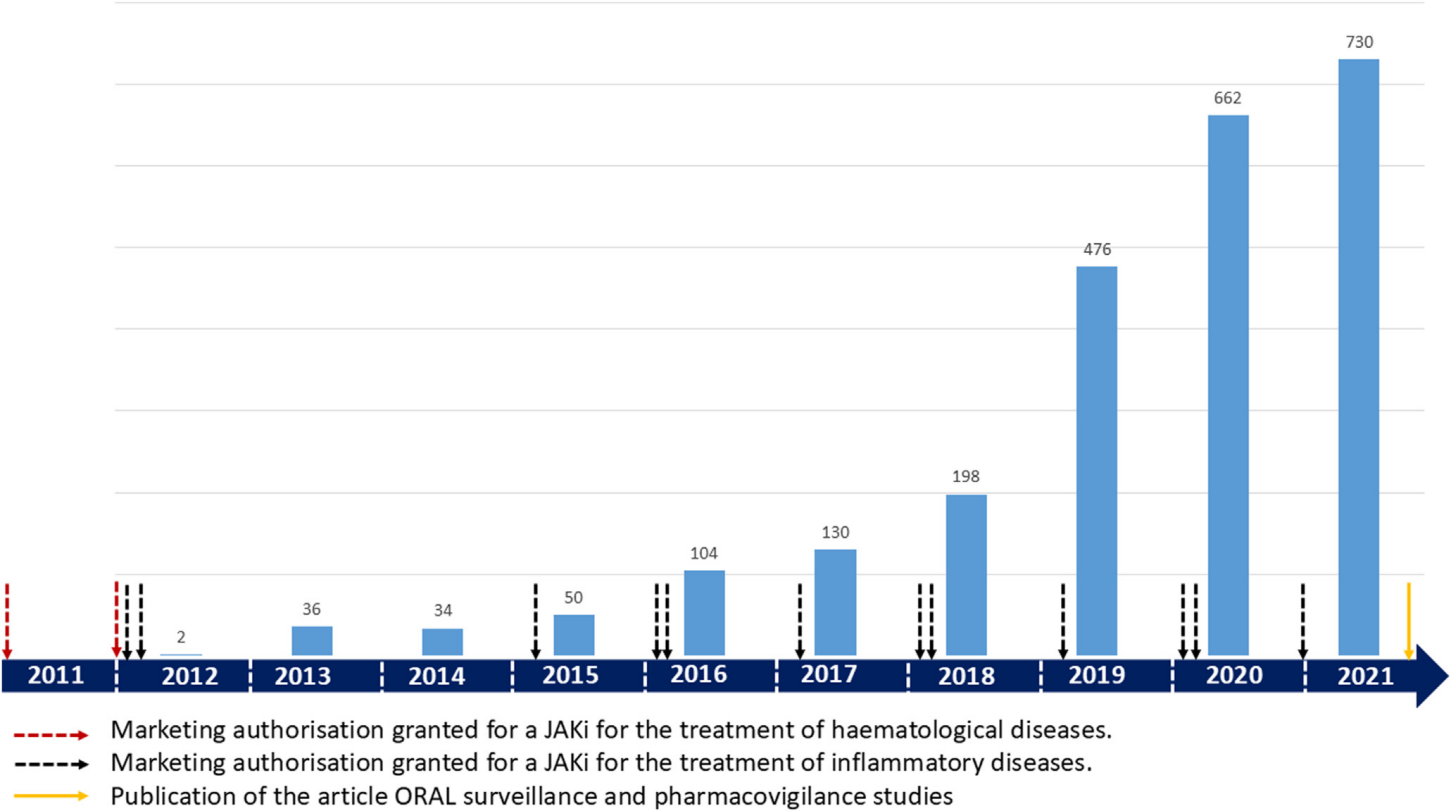
Date of marketing authorizations for the various Janus kinase inhibitors (JAKis) and venous thromboembolic events declared to the Food and Drug Administration Adverse Event Reporting System.

### 3.2 Safety studies (phase IV)

To address the limitations of previous studies, research specifically focusing on safety and examining adverse effects has been conducted. These studies are characterized by longer follow-up durations and larger patient populations. They often extend the follow-up period beyond the initial timeline of the original phase I, II, and III studies, tracking cohorts of patients over several years and recording any adverse events. Although these studies focus solely on patients undergoing JAKi treatment and lack a control group, they are crucial for identifying rare long-term adverse events that may emerge years after treatment initiation. A total of 4 such studies have been identified [[35], [36], [37], [38]] (Supplementary Table S2). Their characteristics are detailed in Table 2. They evaluated tofacitinib and baricitinib in the treatment of RA, AD, and ulcerative colitis. With patient numbers ranging from 1000 to 7000 and a median follow-up of 1.6 to 6.5 years, none of these studies revealed an increased risk of VTE over time. Furthermore, although they included groups of patients receiving different doses of tofacitinib and baricitinib (5/10 mg and 2/4 mg respectively), no dose-dependent difference in risk was observed. However, in the absence of a control group, interpretation of these studies is difficult, and this type of study is of greatest interest for adverse effects occurring with a delay after product from introduction, such as the occurrence of cancer.

**Table 2:** Summary of phase IV studies of JAKi in inflammatory diseases.

Characteristic	Atopic dermatitis (*n* = 1)^a^	Rheumatoid arthritis (*n* = 3)^a^	Ulcerative colitis (*n* = 1)^a^	Overall (*N* = 5)^a^
Study design				
Randomized	0/1	1/3^b^	0/1	1/5
Register	1/1	2/3	1/1	4/5
Participant numbers	2636	4362	944	3770
Median duration (y)	1.60	4.00	6.50	4.00
Drug				
Baricitinib	1/1	1/3	0/1	2/5
Tofacitinib	0/1	2/3	1/1	3/5
Control	0/1	1/3^c^	0/1	1/5
Several JAKi doses	1/1	3/3	1/1	5/5
Increased VTE risk between groups	0/1	1/3^d^	0/1	1/5
Change in VTE incidence over time	0/1	0/3	0/1	0/5

JAKi, Janus kinase inhibitor; VTE, venous thromboembolic event.

a *n*/*N* for categorical variables; median for continuous variables.

b Randomized (1-1-1), open-label.

c Anti-tumor necrosis factor.

d Group-dependent, increase only in the group treated with 10 mg twice daily.

The ORAL Surveillance study, unlike previous studies, is not limited to the follow-up of patients included in phase I to III studies. It is a randomized, noninferiority, postmarketing phase IV study. The primary objective was to assess the risk of major adverse cardiovascular events or cancer in patients with active RA treated with JAKis or anti-TNF. Patients on stable-dose methotrexate (MTX) were randomized in a 1:1:1 ratio to receive either tofacitinib 5 mg or 10 mg twice daily or an anti-TNF agent. With a primary objective focusing on cardiovascular events, participants had to be aged over 50 and have at least 1 cardiovascular risk factor, such as smoking, hypertension, hypercholesterolemia, diabetes, or a history of coronary heart disease. The evaluation of VTE incidence, encompassing PE and DVT, was set as a secondary goal of the research. This study, which tracked 4362 participants over a median duration of 4 years, identified a higher risk of VTE among those administered 10 mg of tofacitinib twice daily compared with those treated with anti-TNF agents. However, this elevated risk was not observed in participants taking a 5 mg dose twice daily. However, a number of limitations have to be pointed: tofacitinib has been granted marketing authorization in Europe at a dose of 5 mg twice daily for the treatment of RA, and not at a dose of 10 mg twice a day. Furthermore, the study was not designed to compare the risk of VTE associated with the different treatment regimens. The VTE risk profile of participants differed between the groups, with 2.3% of patients in the tofacitinib 10 mg twice daily group having a history of VTE, compared with only 1.3% in the 5 mg twice daily group, introducing a potential confounding bias. We did not find any temporal indication between the introduction of JAKis and the occurrence of VTE. In the phase IV studies, the delay between the introduction of JAKis and VTE was not reported. However, these studies, with their aforementioned biases, do not provide definitive confirmation of the associated risk of VTE, and if there is a risk, the patients at risk of VTE are those who would be at risk on JAKis. As a result, more studies, in particular studies in real-life conditions of use, in each indication to take into account the profile of patients and the disease are required.

### 3.3 Real-life studies

Real-life studies offer the advantage of not having the over-selection bias associated with the clinical studies described above. They reflect the real-life use of drugs in all types of patients, making it easier to extrapolate results to the general population. However, the lack of randomization and their often-retrospective design introduce potential biases leading to the use of various statistical techniques such as propensity scoring, matching or multivariate analysis.

Six real-life studies involving a population of about 16,000 patients and focusing exclusively on RA were reviewed. Four studies used propensity scores, 1 self-controlled series, and the last was adjusted with multivariate analysis (Table 3) [23,[39], [40], [41], [42], [43]]. The results were mixed, with 3 studies favoring and 3 opposing an increased risk of thrombosis associated with JAKis. However, the studies in favor of an excess of risk report questionable results. For example, a US cohort study in RA showed an increased risk of VTE in patients receiving baricitinib with an incidence rate ratio of 1.51 (95% CI, 1.10-2.08). However, the overall incidence rate difference, which directly measures the effect of the JAKi in terms of additional cases, was not significant [39]. Another study performed from the Swedish RA cohort found an increased risk of VTE in adjusted analysis in patients treated with JAKis compared with patients treated with anti-TNF [43]. However, this excess risk was statistically significant only for baricitinib (HR, 2.00; 95% CI, 1.41-2.83), with no information on the dose received. The third study, which concluded that there was an excess risk of thrombosis for tofacitinib and baricitinib after their introduction [41], used a self-controlled series comparing VTE in the same patient, whether treated with JAKis or not. In real-life studies, the median time to the occurrence of VTE is reported in only 1 study and was 4.6 months [41]. The intrinsic risk associated with each JAKi has not been assessed. This method enables bias associated with the presence of underlying disease and VTE risk to be controlled. However, a more active disease may increase both the risk of VTE, as seen above, and the likelihood of initiating a second-line treatment such as JAKis, which could contribute to explain the observed excessed risk. Most studies that do not support an increased risk have used statistical methods that are particularly well suited to this research question in order to reduce the risk of confounding bias [23,42,43]. One study in particular used propensity scores, taking into account the time since initiation of treatment with MTX (the reference first-line treatment for RA) [42]. The authors of this study concluded that JAKi is safe in terms of risk of VTE in patients treated for RA, including patients over 65 at high cardiovascular risk (subgroup of the study), echoing the ORAL Surveillance study. The delay from initiation of MTX to inclusion was not addressed in ORAL Surveillance. Finally, although all these studies focus on RA, studies are underway in other indications [44].

**Table 3:** JAKi real-life studies in inflammatory diseases.

Study names	Disease	Methodology	Patient number	Drug	Control	Several JAKi doses	Increased VTE risk between groups
Salinas et al. [39]	RA	Propensity score	16619	Baricitinib	anti-TNF	Yes^a^	Analysis-dependent
Kremer et al. [40]	RA	Propensity score^b^	10357	Tofacitinib	bDMARDs	No	No
Desai et al. [23]	RA	Propensity score	34627	Tofacitinib	anti-TNF	NA^c^	No
Gouverneur et al. [41]	RA	self-controlled case series	5870	Tofacitinib, baricitinib	No	Yes	Yes for both JAKis
Hoisnard et al. [42]	RA	Propensity score	15835	Tofacitinib, baricitinib	Adalimumab	Yes	No
Molander et al. [43]	RA	Adjustment by multivariate analysis	22304	Tofacitinib, baricitinib, upadacitinib	anti-TNF	NA	Yes only for baricitinib

anti-TNF, anti-tumor necrosis factor; bDMARDs, biological disease-modifying antirheumatic drugs; JAKi, Janus kinase inhibitor; NA, not available; RA, rheumatoid arthritis; VTE, venous thromboembolic event.

a But no analyse per dose.

b But VTE assessed in the unmatched population.

c The authors’ conclusions relate only to low dose tofacitinib, with the justification that in RA only low dose tofacitinib is recommended.

## 4 Discussion

### 4.1 Precautionary measure

Based on the evidence reviewed in preceding studies, regulatory authorities such as the FDA and European Medicines Agency have implemented precautionary guidelines in response to initial signals of VTE risk potentially associated with JAKis. These guidelines are intended to carefully weigh the therapeutic benefits of JAKis against their potential risks, especially in high-risk patient populations.

The FDA and the European Medicines Agency have issued alerts and recommendations, respectively, in 2021 and 2023, on the use of JAKis in patients with inflammatory diseases without specific distinction. These recommendations were strongly influenced by findings from the ORAL Surveillance study, which highlighted potential thrombotic risks.

Both agencies specified that JAKis should not be used in patients considered at risk of VTE (such as men aged over 65 or patients with known risk factors for VTE [9,10]). Subsequently, in Europe, some recommendations were published by physicians advocating not to introduce JAKis in inflammatory rheumatic diseases in patients at risk of VTE [45,46].

### 4.2 Revisiting the thrombosis risk: underlying diseases vs JAKis and future study directions

While the ORAL Surveillance trial provides valuable data, it has limitations in specifically addressing the risk of VTE. The study was not designed to assess VTE as a primary outcome, and factors such as prior history of thrombosis were not accounted for. Ideally, a similar study, specifically designed to evaluate thrombotic risk in targeted populations, would be beneficial. However, such studies are costly and challenging to implement. A more realistic alternative would be to promote well-designed registries and real-world studies by molecule and indication, taking into account thrombosis risk factors. While such studies do exist, they do not yet cover all molecules and indications. Furthermore, as these are observational studies, a single study is often insufficient to demonstrate a causal relationship; multiple studies are therefore needed to reach robust conclusions.

Taking into account confounding factors such as disease activity, age, gender, and underlying conditions is essential to better define the thrombotic risk associated with JAKis across different patient profiles. VTE is inherently multifactorial, and the interplay of various contributing factors makes it challenging to isolate the specific impact of JAKis on thrombotic risk.

One factor that remains often underestimated is the baseline disease activity, which can significantly impact thrombotic risk and confound the observed associations. Studies assessing the risk of VTE associated with JAKis do not all converge toward uniform conclusions. Each type of research reveals its own strengths and weaknesses, often influenced by the presence of confounding factors. One particularly notable factor is the underlying disease itself. JAKi is frequently prescribed in contexts with an inherently high risk of VTE, such as myeloproliferative syndromes or inflammatory diseases like inflammatory bowel disease. Analysis of the sites of reported VTE by drug shows that for ruxolitinib (prescribed for myeloproliferative syndromes and not inflammatory diseases), the FDA’s pharmacovigilance data report an increased risk of portal vein thrombosis compared with other adverse effects of ruxolitinib, including other sites of thrombosis [31]. This complication is frequently encountered in myeloproliferative disorders, for which ruxolitinib is indicated [47]. Consequently, it is possible that observed VTE may be attributed to the treatment when they are merely reflections of the underlying disease. Furthermore, although pharmacovigilance has also issued warnings about JAKis used in hematologic diseases, the precautionary measures do not specifically relate to hematologic indications. This may be due to the high thrombotic risk inherently associated with these hematologic diseases, suggesting that thrombosis observed with JAKi treatment in these cases would be attributed to the underlying disease rather than the treatment itself. It is imperative to re-evaluate the risk of VTE in the context of underlying diseases rather than attributing it solely to JAKi treatment. The high thrombotic risk observed in patients on JAKis could very well result from the inherent risk posed by their primary medical conditions. Therefore, a nuanced approach that considers the underlying disease is essential for accurately assessing and managing VTE risk, ensuring that treatment decisions are based on a comprehensive understanding of the patient’s overall health profile.

### 4.3 Thrombosis potential and anti–Janus kinase therapies: unveiling the risks and mechanisms

Although the ORAL Surveillance study suggests a dose-dependent effect for tofacitinib in RA, potentially increasing the risk of VTE, other data are against the hypothesis of a dose effect. Recent research on the use of very high dose JAKis in patients with a genetic immune deficiency with gain of function affecting the JAK-STAT pathway, and accompanied by autoimmune, lymphoproliferative and/or infectious complications, did not reveal any VTE, thus raising the question of the intrinsic risk of VTE linked to inflammatory diseases, irrespective of JAKi and dose treatment [48].

The potential role of JAKis in vascular-related thrombosis warrants attention. Insights can be drawn from studies on the JAK2 tyrosine kinase that results in a valine-to-phenylalanine substitution at position 617 (JAK2 V617F) mutation in endothelial cells, a key player in thrombosis. This mutation causes constitutive activation of the JAK-STAT pathway, leading to altered endothelial behavior such as enhanced proliferation, increased adhesion molecule expression, and a proinflammatory state, creating a thrombogenic environment [49,50]. For example, Sozer et al. [51] demonstrated the JAK2 V617F mutation in endothelial cells from polycythemia vera patients, linking it to prothrombotic phenotypes [51]. Key mechanisms include upregulation of adhesion molecules like P-selectin, which promotes platelet and leukocyte adhesion and thrombus formation [49,50]. Endothelial colony–forming cells (ECFCs), isolated from blood, provide a noninvasive method to study endothelial dysfunction. ECFCs exhibit clonogenic potential, express endothelial markers, and reflect vascular pathology [52,53]. Studies by Yoder et al. [54] and Teofili et al. [55] found JAK2 V617F–positive ECFCs in patients with myeloproliferative neoplasms and thrombotic complications, suggesting a role in vascular events. On these pathophysiological arguments, JAKis are emerging therapies that should have the potential to mitigate vascular inflammation, endothelial prothrombotic activation, and leukocyte-endothelial adhesion. While promising, their impact on thrombosis in myeloproliferative neoplasms requires further translational and clinical validation [5,[56], [57], [58], [59], [60], [61]].

## 5 Conclusion

JAKis have transformed the treatment landscape for various inflammatory and autoimmune diseases, providing substantial therapeutic benefits. As research progresses, additional indications for JAKis, such as lupus, dermatomyositis, sarcoidosis, hemophagocytic lymphohistiocytosis, and immune-checkpoint inhibitor myocarditis, may emerge in the coming years [[62], [63], [64], [65], [66]].

This potential expansion underscores the importance of thoroughly assessing the safety profile of JAKis across diverse diseases. Although some concerns regarding a potential increase in VTE risk have been raised, current evidence remains inconclusive, with real-world studies suggesting that, if a risk exists, it may be minimal or confined to specific patient profiles. Given the multifactorial nature of VTE, more targeted research is needed to disentangle the influence of JAKis from underlying disease activity and other risk factors. Dedicated studies, tailored to specific indications and patient populations, are essential to refine our understanding of VTE risk and ensure safe, optimized use of JAKis in clinical practice.
