# Supplementary material for: Janus kinase inhibitor treatment for inflammatory diseases: excess or no excess risk of venous thromboembolism?
Source: Res Pract Thromb Haemost. 2024 Dec 31;9(1):102667. doi: 10.1016/j.rpth.2024.102667 (PMC11840193; doi:10.1016/j.rpth.2024.102667)
Supplement: Supplementary material [file mmc1.docx]

**Supplementary**

1. Supplementary Table S1: Clinical studies phase I, II et III

| Study name | Disease | Phase RCT | Duration  (week) | Drug | JAKi^1^  Number group | Disease severity | average number per group | JAKi^1^  VTE^2^ count | Control VTE^2^ count | JAKi^1^ ATE^3^ count | Control ATE^3^ count | **OR^4^ TE^5^** | **OR^4^ VTE^2^** | Outcome VTE**^2^** | exclusion of patients with VTE^2^ history |
| --- | --- | --- | --- | --- | --- | --- | --- | --- | --- | --- | --- | --- | --- | --- | --- |
| RA Beam, 2017 | RA | III | 24 | Baricitinib | 1 | Moderately to severely | 487,5 | 0 | 0 | 1 | 0 | 3.01 [0.12; 74.13] |  | No | Yes |
| RA Build, 2017 | RA | III | 24 | Baricitinib | 2 | Moderately to severely | 228 | 0 | 0 | 0 | 2 | 0.1 [0.0; 2.08] |  | No | Yes |
| RA Beacon, 2016 | RA | III | 24 | Baricitinib | 2 | Moderately to severely | 176 | 0 | 0 | 2 | 0 | 2.52 [0.12; 52.88] |  | No | Yes |
| RA Begin, 2017 | RA | III | 52 | Baricitinib | 1 | Mild to severe | 213 | 0 | 1 | 0 | 0 | 0.32 [0.12; 8.0] | 0.32 [0.01; 8.0] | No | Yes |
| RA Balance, 2020 | RA | III | 52 | Baricitinib | 1 | Moderately to severely | 116 | 0 | 0 | 0 | 0 |  |  | No | No |
| FINCH 1, 2021 | RA | III | 24 | Filgotinib | 2 | Moderately to severely | 477 | 1 | 2 |  |  | 0.25 [0.02; 2.74] | 0.25 [0.02; 2.74] | No | No |
| FINCH 2, 2019 | RA | III | 24 | Filgotinib | 2 | Moderately to severely | 150 | 1 | 0 | 1 | 0 | 2.49 [0.12; 52.14] | 1.49 [0.06; 36.73] | No | No |
| FINCH 3, 2021 | RA | III | 52 | Filgotinib | 2 | Active disease | 346 | 0 | 2 | 3 | 2 | 0.5 [0.11; 2.24] | 0.13 [0.01; 2.78] | No | No |
| RAJ3, 2019 | RA | III | 12 | Peficitinib | 2 | Active disease | 102 | 0 | 0 | 0 | 0 |  |  | Yes | Yes |
| ORAL Scan, 2019 | RA | III | 12 | Tofacitinib | 2 | NR | 266 | 0 | 0 | 0 | 0 |  |  | No | unclear |
| ORAL Sync, 2013 | RA | III | 48 | Tofacitinib | 2 | Active disease | 264 | 0 | 0 | 2 | 0 | 1.26 [0.06; 26.44] |  | No | unclear |
| ORAL Step, 2013 | RA | III | 12 | Tofacitinib | 2 | Moderately to severely | 133 | 0 | 0 | 0 | 0 |  |  | Yes | unclear |
| ORAL Solo, 2012 | RA | III | 12 | Tofacitinib | 2 | Active disease | 203 | 0 | 2 | 0 | 0 | 0.05 [0.0; 1.03] | 0.05 [0.0; 1.03] | Yes | No |
| Kremer, 2009 | RA | II | 6 | Tofacitinib | 1 | Active disease | 63 | 0 | 0 | 0 | 0 |  |  | No | No |
| SELECT Sunrise, 2020 | RA | III | 12 | Upadacitinib | 2 | Moderately to severely | 49 | 0 | 0 | 0 | 0 |  |  | No | No |
| SELECT Compare, 2019 | RA | III | 26 | Upadacitinib | 1 | Active disease | 651 | 2 | 1 | 0 | 2 | 0.67 [0.11; 4.0] | 2.0 [0.18; 22.14] | No | No |
| SELECT Next, 2018 | RA | III | 12 | Upadacitinib | 2 | Moderately to severely | 220 | 0 | 0 | 1 | 0 | 1.51 [0.06; 37.27] |  | No | No |
| SELECT Beyond, 2018 | RA | III | 12 | Upadacitinib | 2 | Mild to moderate | 166 | 1 | 0 | 1 | 0 | 2.59 [0.12; 54.21] | 1.55 [0.06; 38.2] | No | No |
| BALANCE I, 2016 | RA | II | 12 | Upadacitinib | 1 | Moderately to severely | 56 | 0 | 0 | 0 | 0 |  |  | No | No |
| BALANCE II, 2016 | RA | II | 12 | Upadacitinib | 2 | Mild to moderate | 50 | 0 | 0 | 1 | 0 | 1.54 [0.06; 38.44] |  | Yes | No |
| Mease, 2021 | PsA | II | 16 | Deucravacitinib | 2 | Moderately to severely | 68 | 0 | 1 | 0 | 0 | 0.16 [0.01; 3.95] | 0.16 [0.01; 3.95] | No | No |
| EQUATOR, 2018 | PsA | II | 12 | Filgotinib | 1 | Moderately to severely | 66 | 0 | 0 |  |  |  |  | Yes | No |
| OPAL Beyond, 2017 | PsA | III | 12 | Tofacitinib | 2 | Moderately to severely | 131 | 0 | 0 | 0 | 0 |  |  | No | No |
| OPAL Broaden, 2017 | PsA | III | 12 | Tofacitinib | 2 | Active disease | 105 | 0 | 0 | 0 | 0 |  |  | No | No |
| SELECT PsA 1, 2021 | PsA | III | 24 | Upadacitinib | 2 | Active disease | 427 | 1 | 1 |  |  | 0.5 [0.03; 8.06] | 0.5 [0.03; 8.06] | No | No |
| SELECT PsA 2, 2021 | PsA | III | 24 | Upadacitinib | 2 | Active disease | 214 | 1 | 0 | 1 | 0 | 2.49 [0.12; 52.0] | 1.49 [0.06; 36.67] | No | No |
| TORTUGA, 2018 | AS | II | 12 | Filgotinib | 1 | Active disease | 58 | 1 | 0 | 0 | 0 | 3.05 [0.12; 76.48] | 3.05 [0.12; 76.48] | Yes | No |
| Deodhar, 2021 | AS | III | 16 | Tofacitinib | 2 | Active disease | 135 | 0 | 0 | 0 | 0 |  |  | Yes | No |
| Van Der Heijde, 2017 | AS | III | 12 | Tofacitinib | 2 | Active disease | 52 | 0 | 0 | 0 | 0 |  |  | No | No |
| SELECT Axis 1, 2019 | AS | III | 14 | Upadacitinib | 1 | Active disease | 93,5 | 0 | 0 | 0 | 0 |  |  | No | No |
| SELECTION, 2017 | UC | III | 10 | Filgotinib | 2 | Moderately to severely | 449 | 1 | 0 | 0 | 0 |  |  | No | No |
| OCTAVE Induction 1, 2017 | UC | III | 8 | Tofacitinib | 1 | Moderately to severely | 299 | 0 | 0 | 2 | 0 | 1.29 [0.06; 27.06] |  | No | No |
| OCTAVE Induction 2, 2017 | UC | III | 8 | Tofacitinib | 1 | Moderately to severely | 271 | 0 | 0 | 0 | 0 |  |  | No | No |
| Sandborn, 2012 | UC | II | 12 | Tofacitinib | 1 | Moderately to severely | 41 | 0 | 0 | 0 | 1 | 0.47 [0.02; 11.96] |  | Yes | No |
| U-ACHIEVE, 2020 | UC | II | 8 | Upadacitinib | 3 | Moderately to severely | 51 | 2 | 0 | 0 | 0 | 1.5 [0.07; 31.7] | 1.5 [0.07; 31.7] | No | No |
| Panes, 2017 | Crohn | II | 8 | Tofacitinib | 2 | Moderately to severely | 87 | 0 | 0 | 0 | 0 |  |  | No | Yes |
| CELEST, 2020 | Crohn | II | 16 | Upadacitinib | 2 | Moderately to severely | 36 | 0 | 0 | 1 | 0 | 1.57 [0.06; 39.57] |  | Yes | No |
| JADE Compare, 2021 | AD | III | 16 | Abrocitinib | 2 | Moderately to severely | 198 | 0 | 0 | 0 | 0 |  |  | No | Unclear |
| JADE Teen, 2021 | AD | III | 12 | Abrocitinib | 2 | Moderately to severely | 95 | 0 | 0 | 0 | 0 |  |  | Yes | Unclear |
| JADE Mono 1, 2020 | AD | III | 12 | Abrocitinib | 2 | Moderately to severely | 129 | 0 | 0 | 0 | 0 |  |  | No | Unclear |
| JADE Mono 2, 2020 | AD | III | 12 | Abrocitinib | 2 | Moderately to severely | 130 | 0 | 0 | 0 | 0 |  |  | No | Unclear |
| Gooderham, 2019 | AD | Phase II RCT | 12 | Abrocitinib | 2 | Moderately to severely | 56 | 1 | 0 | 0 | 0 | 1.53 [0.06; 38.26] | 1.53 [0.06; 38.26] | Yes | No |
| BREEZE AD1, 2020 | AD | III | 16 | Baricitinib | 3 | Moderately to severely | 156 | 0 | 0 | 0 | 0 |  |  | No | Yes |
| BREEZE AD2, 2020 | AD | III | 16 | Baricitinib | 3 | Moderately to severely | 154 | 0 | 0 | 0 | 0 |  |  | No | Yes |
| BREEZE AD5, 2021 | AD | III | 16 | Baricitinib | 2 | Moderately to severely | 147 | 0 | 0 | 0 | 0 |  |  | No | Yes |
| BREEZE AD7, 2020 | AD | III | 16 | Baricitinib | 2 | Moderately to severely | 110 | 1 | 0 | 0 | 0 | 1.5 [0.06; 38.26] | 1.5 [0.06; 38.26] | No | Yes |
| Zhao, 2021 | AD | II | 12 | Ivarmacitinib | 2 | Moderately to severely | 35 | 0 | 0 | 0 | 0 |  |  | No | Yes |
| MEASURE Up 1, 2021 | AD | III | 16 | Upadacitinib | 2 | Moderately to severely | 282 | 0 | 0 | 0 | 0 |  |  | No | No |
| MEASURE Up 2, 2021 | AD | III | 16 | Upadacitinib | 2 | Moderately to severely | 279 | 0 | 1 | 0 | 0 | 0.17 [0.01; 4.08] | 0.17 [0.01; 4.08] | No | No |
| AD UP, 2021 | AD | III | 16 | Upadacitinib | 2 | Moderately to severely | 267 | 0 | 0 | 0 | 1 | 0.11 [0.0; 2.8] |  | No | No |
| Guttman-Yassky, 2020 | AD | II | 16 | Upadacitinib | 2 | Moderately to severely | 42 | 0 | 0 | 0 | 0 |  |  | No | No |
| Schmieder, 2018 | Plaque psoriasis | II | 4 | Abrocitinib | 1 | Moderately to severely | 15 | 0 | 0 | 0 | 1 | 0.29 [0.01; 7.74] |  | No | No |
| Forman, 2020 | Plaque psoriasis | II | 12 | Brepocitinib | 1 | Moderately to severely | 26 | 0 | 0 | 0 | 0 |  |  | Yes | No |
| Papp, 2018 | Plaque psoriasis | II | 12 | Deucravacitinib | 2 | Moderately to severely | 45 | 0 | 0 | 0 | 0 |  |  | Yes | Unclear |
| Zhang, 2017 | Plaque psoriasis | III | 16 | Tofacitinib | 2 | Moderately to severely | 89 | 0 | 0 | 0 | 0 |  |  | Yes | No |
| OPT Pivotal 1, 2017 | Plaque psoriasis | II | 16 | Tofacitinib | 2 | Moderately to severely | 300 |  |  |  |  |  |  | Yes | No |
| OPT Pivotal 2, 2017 | Plaque psoriasis | II | 16 | Tofacitinib | 2 | Moderately to severely | 320 |  |  |  |  |  |  | Yes | No |
| OPT Compare, 2015 | Plaque psoriasis | III | 12 | Tofacitinib | 2 | Moderately to severely | 255 | 0 | 0 | 1 | 0 | 0.49 [0.02; 12.1] |  | No | No |
| Wallace, 2018 | SLE | II | 24 | Baricitinib | 2 | Active disease | 105 | 1 | 0 | 0 | 0 | 1.52 [0.06; 37.58] | 1.52 [0.06; 37.58] | No | Yes |
| Werth, 2021 | SLE | II | 12 | Filgotinib | 1 | Moderately to severely | 13 | 0 | 0 | 0 | 0 |  |  | No | No |
| Hasni, 2021 | SLE | I | 12 | Tofacitinib | 1 | Mild to moderate | 15 | 0 | 0 | 0 | 0 |  |  | Yes | No |
| TACOS, 2021 | UC | III | 3 | Tofacitinib | 2 | Severe | 52 | 1 | 0 | 0 | 0 |  |  | Yes | Yes |
| ^1^JAKI: Janus kinas inhibitor  ^2^VTE: Thromboembolic venous event  ^3^ATE: Thromboembolic arterial event.  ^4^OR: odds ratio  ^5^TE: Thromboembolic event  SAE: Severe adverse effect | | | | | | | | | | | | | |  |  |

Supplementary Table S2: Clinical studies phase IV

| Study name | Disease | Design | Participant number | Duration  (Years) | Drug | Control | Several JAKi^1^ doses | Increased VTE^2^ risk between groups | Change in VTE^2^ incidence over time |
| --- | --- | --- | --- | --- | --- | --- | --- | --- | --- |
| ORAL surveillance, 2022 | RA | Randomized (1-1-1), open-label | 4362 | 4 | Tofacitinib | Yes^3^ | Yes | group-dependent^4^ | No |
| ORAL sequel, | RA | Register | 7061 | 3,1 | Tofacitinib | No | Yes | No | No |
| RA-BEYOND | RA | Register | 3770 | 4,6 | Baricitinib | No | Yes | No | No |
| BREEZE | AD | Register | 2636 | 1,6 | Baricitinib | No | Yes | No | No |
| OCTAVE OPEN | Ulcerative colitis | Register | 944 | 6,5 | Tofacitinib | No | Yes | No | No |
| ^1^JAKI: Janus kinas inhibitor  ^2^VTE: Thromboembolic venous event  ^3^TNF inhibitor  ^4^Increase only in the group treated with 10 mg twice daily. | | | | | | | | | |
